# Prevalence of Intestinal Schistosomiasis and Associated Factors among School Children in Wondo District, Ethiopia

**DOI:** 10.1155/2020/9813743

**Published:** 2020-03-24

**Authors:** Mustafa Geleto Ansha, Kemal Ahmed Kuti, Ephrem Girma

**Affiliations:** ^1^Department of Public Health, Madda Walabu University, Goba, Ethiopia; ^2^Department of Communicable Diseases, Wondo Woreda Health Office, Intaye, Goba, Ethiopia

## Abstract

**Background:**

Human schistosomiasis is one of the neglected tropical diseases caused by *Schistosoma mansoni*. Children in the developing countries live in areas with poor sanitation and most often spend time swimming or bathing in the water bodies contaminated with cercariae, the infective stages of schistosomiasis, which results in growth retardation and poor school performance. Thus, having effective control of the disease requires assessment of prevalence and risk factors.

**Objective:**

This study was aimed at assessing the prevalence of *S. mansoni* and its associated factors among primary schoolchildren in Wondo district, West Arsi Zone, Ethiopia, 2018.

**Methods:**

A cross-sectional study was carried out between January and March 2018. Stool samples from 298 schoolchildren, who were selected by simple random sampling, were examined for the ova of *S. mansoni* using Kato–Katz technique. Information on sociodemographic factors and other risk factors was obtained using questionnaires. The data were cleaned, coded, and entered into SPSS 22.0 statistical software and analyzed. Bivariable and multivariable logistic regression analysis was done to identify factors associated with *S. mansoni* infection. Adjusted odds ratio (AOR) with 95% confidence interval (CI) was calculated, and the level of significance was declared at *p* values of less than 0.05. The result was presented using tables, figures, and text form.

**Result:**

A total of 298 study participants were involved in this study resulting in a response rate of 96.4% (298/309). The prevalence of *S. mansoni* infection was 11.4% (34/298). The prevalence was 8% (12/140) among males while it was 13% (22/158) among females. Swimming in rivers or ponds (AOR: 9.592; 95% CI: 1.972–46.655; *P*=0.005), latrine availability at household level (AOR: 0.075; 95% CI: 0.13–0.422; *P*=0.003), and awareness about schistosomiasis (AOR: 0.058; 95% CI: 0.004–0.409; *P*=0.007) were the factors independently associated with *S. mansoni* infection at *p* value < 0.05.

**Conclusion:**

The prevalence of *S. mansoni* was moderate as per the World Health Organization standard, since it was in the range of 10%–50%. This implies that schistosomiasis is still among major health problems. Thus, intensified effort is needed to address risk factors contributing to infection and control disease. Additionally, biannual mass drug administration with praziquantel is required according to the WHO standard.

## 1. Introduction

Human schistosomiasis, otherwise called bilharzia, is a fresh water snail transmitted intravascular debilitating disease that results from infection by trematode worms known as *Schistosoma*, which lives in the bloodstream of humans [1]. It is one of the neglected tropical parasitic diseases (NTDs) and causes severe morbidity and mortality among susceptible segments of the population. In tropical and subtropical countries, it has high public health importance and socioeconomic impacts [2, 3].

Schistosomiasis is mainly caused by three different species of blood-dwelling fluke worms of the genus *Schistosoma*, namely, *S. haematobium*, which causes urinary schistosomiasis, and *S. mansoni* and *S. japonicum*, which cause intestinal schistosomiasis. Clinical manifestations of schistosomiasis are associated with the species-specific ova and the burden of infection [4]. Among the three species, *S. mansoni* is the chief cause of clinical abnormalities such as hepatomegaly, splenomegaly, and periportal fibrosis in various sub-Saharan African countries. In Ethiopia, *S. mansoni* is transmitted mainly by *Biomphalaria sudanica* (*B. sudanica*) and *Biomphalaria pfeifferi* (*B. pfeifferi*) snail intermediate hosts. *B. sudanica* is distributed in few water bodies in southern part of the country, whereas *B. pfeifferi* has a wider geographical distribution in Ethiopia [5].

Humans are usually infected by cercarial invasion through the skin when they come into contact with contaminated fresh water during daily life. In the endemic areas, children, women, fishermen, and farmers in irrigation channels are often infected with schistosomiasis [6]. Children in the developing countries live in areas with poor sanitation and most often spend time swimming or bathing in the water bodies contaminated with cercariae, the infective stages of schistosomiasis. The nonhygiene and playing behavior in water bodies increase the risk of being infected by *S. mansoni *[4].

Though schistosomiasis is rarely fatal, it causes long-term morbidity such as anaemia which results from bleeding from the urinary and intestinal tracts due to worm invasion and movements; iron deficiency is also an outcome of this disease. In this regard, it is similar to nutritional impairment such as nutrient malabsorption and digestive disorder. This chronic inflammatory process is associated with long-term schistosomiasis and contributes to anemia and undernutrition, which, in turn, can lead to growth stunting, learning disabilities, school absenteeism and higher dropout rates, poor school performance, poor work productivity, and continued poverty [7].

The prevalence and morbidity of schistosomiasis is highest among school-age children due to high exposure to infested water bodies. Growth retardation and poor school performance are adverse effects of the disease besides its clinical manifestation and complication [8]. On top of that, its negative impact on school performance and the debilitation it causes when not treated demoralize both social and economic development in endemic areas [4]. Reasons for the persistence of the disease in spite of prolonged control and preventive efforts include wide distribution of the intermediate host, migration, the dependency of many poor populations in both rural and urban areas on *Schistosoma*-infested water sources for their domestic, occupational and recreational needs, lack of sanitation, scarcity of portable water, and deficiencies in preventive and curative health services [9]. In the past, the preventive measures of schistosomiasis was largely focused on decreasing or interrupting transmission of the infection; however, such measures have not been continued due to high operation cost/difficult implementation logistics [4].

Even though schistosomiasis is a public health problem in Ethiopia, estimates of its prevalence vary widely and the reports about *S. mansoni* occurrence vary and lack consistency. Thus, there is need for updated information on the extent of disease burden in the community and factors associated with schistosomiasis infection at the district and subdistrict level to facilitate effective prevention and control programs at the local level. Hence, this study assessed the situation of the intestinal schistosomiasis among schoolchildren of Wondo district, West Arsi Zone, Southern Ethiopia.

## 2. Materials and Methods

### 2.1. Study Design and Setting

A cross-sectional study was conducted between January and March 2018 among students of three primary schools, namely, Busa, Intaye, and Shasha in Wondo district, West Arsi Zone, Oromia Regional State, Ethiopia. The district is located 07°05′35″N and 038°36′66″E about 271 km away from the capital city of the country and 19 km away from Shashamane town. The district is located at an altitude ranging from 1700 to 2300 m above sea level.

It is generally characterized by warm climate with a mean annual maximum temperature of 19°C and a mean annual minimum temperature of 17°C. The annual average rainfall is 1244 mm. In the district, there are different water sources which the population frequently use for domestic purposes and thus could be potential risk factors for infection with *S. mansoni.* The total population of the district was 112128 out of which 55503 (49.5%) were male and 56625 (50.5%) were female, and there were 11 primary schools (one per kebele) with a total number of 10494 (5264 male and 5230 female) students enrolled during 2018.

### 2.2. Study Participants

All children enrolled to primary schools during 2018 academic year in the selected three schools of Wondo district, whose age was 5–19 years and who were in school during the study period, were included in this study. However, schoolchildren who were clinically ill at the time of recruitment were excluded.

## 3. Sample Size Determination

The sample size was calculated using a single proportion formula with the following assumptions: at 95% confidence interval (Z$\overset{´}{\alpha }$/2 = 1.96), an expected prevalence of 24% taken from Manna district of Jimma with similar geographical characteristics with the study area [2, 3], 5% marginal error, and a possible nonresponse rate of 10%. Accordingly, *n* = [*Z* 1 − *a*/2] 2 *P* (1 − *p*]/d2 *n* = (1.96/0.05)2 *∗* 0.24 *∗* (1 – 0.24) = 280.29. Adding nonresponse rate, final sample size, *n*, was 309.

## 4. Sampling Technique and Procedure

**Figure 1:**
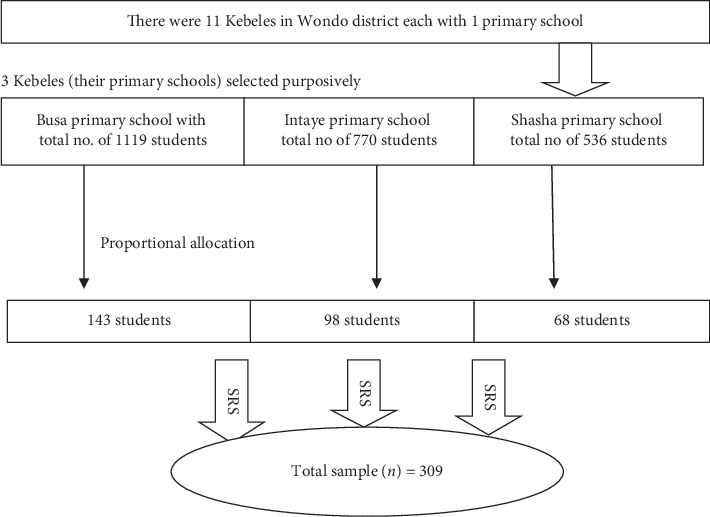
Sampling procedure for assessment of prevalence and factors associated with intestinal schistosomiasis among school-age children in Wondo district, Ethiopia, 2018.

The study area, Wondo district, has 11 subdistricts known as kebele in Ethiopia. This study was conducted in three of these kebeles which were purposively selected because of the presence of intestinal schistosomiasis as evidenced by activity reports from health institutions of the district. Then, the total sample, 309, was proportionally allocated to the primary schools found in the selected kebeles, namely, Busa, Intaye, and Shasha primary schools. Finally, an individual student was selected by simple random sampling technique using tables of random number from sampling prepared using students' record of each school (Figure 1).

## 5. Data Collection Tools/Instruments

A structured questionnaire was developed from review of various related literatures in English. It consists of the parts that address sociodemographic information, hygiene and sanitation, water contact behaviour, and awareness about schistosomiasis. It was translated into Afan Oromo, a local language of the study area. It was retranslated to English to check for consistency of the two versions. It was pretested before the actual data collection on 5% of the total sample size, and modifications were made. Interview data on sociodemographic information, hygiene and sanitation, water contact behaviour, and awareness about schistosomiasis were collected using a questionnaire through a face to face interview. Then, the study participants were linked to laboratory personnel for provision of stool sample at the end of the interview.

## 6. Data Collection Methods

Stool samples were examined using Kato–Katz technique. Materials used were Kato set (template with holes, screen, nylon or plastic, and plastic spatula), newspaper or glazed tile, microscope slides, cellophane as cover slip, soaked in glycerol-malachite green solution, fresh stool, and gloves [10].

A plastic sheet and applicator stick were given to the study participants to bring sizable stool. The slides were labelled using the identification number of the participants and the school code. A single Kato–Katz thick smear per stool sample was prepared from a single stool using a template delivering 41.7 mg of faeces, and stool microscopy was done systematically by the experienced laboratory technician. A multiplication factor of 24 was used to estimate the number of eggs per gram of stool. The intensity of *S. mansoni* infections was categorized as light, moderate, or heavy-infection intensities based on the number of egg counts as per the cut offs described by the WHO [11].

To assure the quality of laboratory procedure, all the reagents, chemicals, and kits were checked before processing and examination of samples by laboratory technicians who have considerable experience in preparing Kato–Katz smears and microscopy of stool samples. The samples were checked for labelling immediately as submitted by the participants, and 10% of the slides were randomly selected and reexamined by a laboratory technician who did not previously examine the slides. The interview data were checked for incompleteness or misclassification, and corrective measures were taken immediately before leaving the study sites.

## 7. Data Processing and Analysis

Data were cleaned, coded, entered, and analyzed using SPSS 22.0 software. Descriptive statistics was used to give a clear picture of background variables. The frequency distribution of both dependent and independent variables was presented. Factors associated with infection of *S. mansoni* were analyzed using binary and multiple logistic regressions to determine independent risk factors. Adjusted odds ratio and its 95% confidence interval were used to present the result regarding association. A *P* value of less than 0.05 was considered to declare the level of statistical significance.

## 8. Ethical Consideration

Ethical approval was obtained from the Ethical Review Board of Oromia Regional Health Bureau under the research ethical clearance code no. ORHB/ERB-108/2018. Before starting the actual data collection, permission to conduct the study was obtained from both district health office and education office. Verbal consent was obtained from students before collecting stool specimens. Additionally, after explaining the importance of the study, informed written consent was obtained from the study participants' parents/guardians, and those children who were positive for *S. mansoni* were linked to district health office for treatment according to the national protocol with a single dose of praziquantel (40 mg/kg body weight).

## 9. Results

### 9.1. General Characteristics of Study Population

Two hundred and ninety-eight schoolchildren (47% males and 53% females) aged between 7 and 19 years, with a mean age of 11.49 and standard deviation of 2.39 have participated in this study. Among these, 150 (50.3%) were from Busa, 40 (13.4%) from Shasha, and 108 (36.2%) from Intaye kebele. The majority of the study participants' (63.1%) age falls between 10- and 14-year age groups.

One out of five children's parents had no formal education, while the rest have primary to tertiary level educational status. More than 70% of children's parents were farmers, whereas 17%, 2%, and 7% were businessperson, government employee, and daily labourer, respectively. Three out of four students' parents had access to pipe water supply while the rest got drinking water from other unprotected sources.

All schools and the majority of students' households had latrine; only 9% HHs lack latrine, and the students of those HHs use neighbours' latrine and field to defecate. In these kebeles, seven local flowing rivers were identified, and almost 93% schoolchildren had contact experience to these rivers; 70% of them had three times per week for playing and swimming in the water streams and pools (Table 1).

## 10. Prevalence and Risk Factors of Schistosomiasis

Thirty-four (11.4%) students were found infected with *S. mansoni*; mean intensity of infection was 25.79 eggs/gram of stool. The prevalence was highest in students from Shasha (15.0%), followed by Intaye (13.0%) and Busa (9.3%) (Table 2).

**Figure 2:**
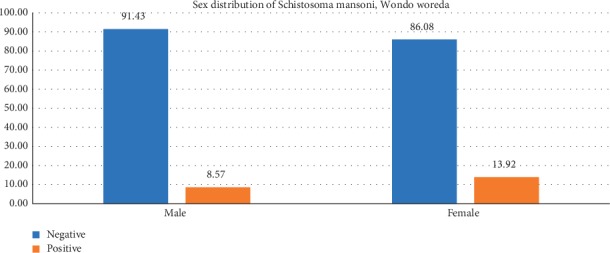
Sex distribution of *S. mansoni* among Wondo district schoolchildren who participated in this study (*n* = 298).

High prevalence of infection detected among females than males (13.9% vs 8.6%) (Figure 2). However, the difference was not statistically significant (*x*2 = 2.104; d*f*; 1 *P*=0.147). Similarly, the prevalence was higher among the age ranges of 10–14 years (12.3%) followed by age 5–9  ears (10.5%) and 15–19 years (8.8%), but found statistically not significant (Table 3).

The highest prevalence and intensity of infection with the disease was observed among students who reported swimming experience (14%), followed by those who reported going to rivers for wash (9%) and herd cattle in the water fields. Students whose parents were farmers were found to have a high prevalence of infection and intensity of infection (Table 4).

Though some of the interviewed students were aware of the disease, most of them did not know about schistosomiasis or its transmission mode. In the result, the disease was found to be higher among the group who had awareness on disease cause and transmission (34.8%) than those who had no any awareness on the disease with 9.5%. High prevalence (27.3%) was found among those who use rivers as water source than other sources followed by spring (12.8%), pipe water (11.2%), and deep well (4.2%).

On the other hand, those who use unsafe sources for drinking water have high prevalence of *S. mansoni* (12.2%) than those drink pipe water (11.2%). Additionally, those living in the HHs without latrine have high prevalence (33.3%) compared to those with latrine (9.2%) (Table 5).

Results of the bivariate analysis for the association of schistosomiasis showed that water contact experience, swimming, awareness about diseases, and lack of latrine are associated with *S. mansoni* prevalence (Table 5).

The risk factors swimming practice in rivers/ponds (AOR = 9.592; 95% CI: 1.972–46.655; *P*=0.005), household latrine (AOR = 0.075; 95% CI: 0.13–0.422; *P*=0.003), and awareness about schistosomiasis (AOR = 0.058; 95% CI: 0.004–0.409; *P*=0.007) were independent predictors for *S. mansoni* infection (*P* value < 0.05) (Table 6).

## 11. Discussion

The prevalence of *S. mansoni* was determined by collecting stool from schoolchildren and looking for ova of parasites by using Kato–Katz technique. Despite the limitation in its sensitivity, the Kato-Katz technique was a widely used and cost-effective method for both diagnostic and survey. Additionally, only this technique is available and practiced in the study area. Accordingly, the prevalence of *S. mansoni* infection in this study was found (11.4%). The finding of this study was much lesser than a prevalence (81.3%) of *S. mansoni* infection observed among Demba Girara primary schoolchildren of Damot Woide district, Wolaita Zone [5] and primary schoolchildren in Sanja area (82.8%), Amhara region [8], Ethiopia. In a cross-sectional study carried out in Zarima town, Northwest Ethiopia among 319 elementary schoolchildren revealed a higher prevalence of 37.9% *S. mansoni* infection [13]. The low prevalence of *S. mansoni* in the present study might be due to presence of potable water in the study area. Inhabitants of the area largely depend on public pipelines, for indoor and outdoor consumptions. On the other hand, this finding was higher than reports from previous investigations conducted in Ethiopia including Tigray (5.95%) [14] and Jimma (2.1%) [15]. The difference might be due to long time endemicity of the parasite in these study areas with the altitude differences.

As indicated in this study, the prevalence of *S. mansoni* was found to be higher among the girls than their counterparts. This study also showed that the mean intensity of infection was higher in girls compared to boys and significantly higher infection rates among females have been also reported elsewhere in Kenya and Zanzibar [16]. However, contrary with the findings conducted in some parts elsewhere in Africa [4, 17, 18], these studies imply higher infection rates and infection intensities in girls. This may be because of high contact of girls with water during fetching and washing clothes. The snail transmitting cercaria prefers to live in fresh water which is mostly utilized for washing clothes and drinking. Moreover, in the present study, highest prevalence and intensity of *S. mansoni* infection were found in the age group 10–14 years. These findings are similar to studies conducted in Jiga, northwestern Ethiopia [8, 13] and Nigeria [19]. High prevalence in this age group may be related to playing and swimming interests in those age groups. Most of early adolescents have swimming habit than any other age groups.

In the present study, the absence of a functioning toilet in the house was significantly associated with the prevalence of schistosomiasis, revealing children whose family has no latrine were almost ten times more likely exposed to the infection than who had latrine. Thus, open defecation is still contributing to transmission of infection and provide information for enhancing open defecation free. With regard to awareness on the infection, it was found the odd of having infection among those who had awareness on the transmission was 38% less likely than that of counterparts.

In the finding, it was also revealed that children who swim in rivers had almost 10 times more likely of being infected with *S. mansoni* than those who did not have a swimming habit. This result was higher as compared to a study carried out at two primary schools in Saja, which is 3.2 times higher exposure in swimming practice [11]. The increased result might be obtained at Wondo due to the presence of many rivers in the locality. The result also supports that those rivers are infected with the cercaria, which gives the clues for district health education and awareness creation focus. Majorities of infection intensities and the mean intensity of infection were low. Meanwhile, this study revealed that *S. mansoni* infections were predominantly light (11.1%) followed by moderate (0.3%) and no heavy infections in the study population.

The infection intensity in the study indicated low infection levels comparable with findings from Timuga and Waja from Tigray [11], Wollega [20] in Ethiopia that had moderate infection intensities. These differences in the prevalence of *S. mansoni* may be ascribed to altitude differences, availability of streams, rivers, or ponds, the frequency of students' contact with water bodies, and playing habits of schoolchildren.

This study employed Kato–Katz technique which is less sensitive in identifying ova of *Schistosoma*, but it is the only available technique in the area and currently utilized for diagnostic purpose.

The prevalence of *S. mansoni* among schoolchildren in the study district was classified under moderate prevalence under the WHO *Schistosoma* Endemicity lCassification. In particular, the disease was higher among the age groups of 10–14 years though all age groups of participants infected showed that more attention is needed for this age group. This is an indication that younger children were more exposed to the source of infection through engagement of activities such as open-field defecation and exposure activities such as washing, swimming, and bathing in the water bodies that favored transmission of schistosomiasis. As the WHO recommended for moderate prevalence, the West Arsi Zone health office and Wondo district health office should carry out a biannual MDA with praziquantel.

There was a statistically significant association between the prevalence of schistosomiasis, water contact practice, and lack of latrine. The district health office and water and sanitation office shall come up with strategies for ensuring that the community uses safe water for domestic use and latrine utilization in the area.

Awareness about diseases also has association with the prevalence of the diseases. This may be due to prior exposure to the infection, which helps to improve health seeking behavior and early treatment. Wondo district health office and respective health facilities should provide regular health education for students and community at large on the mode of transmission, prevention, and control of schistosomiasis infection.

## Figures and Tables

**Table 1:** Sociodemographic characteristics of schoolchildren in three primary schools of Wondo district, West Arsi Zone, South Ethiopia, 2018 (*n* = 298).

Characteristics	Number	(%)
*Sex*		
Male	140	47.0
Female	158	53.0

*Age groups (years)*		
5–9	75	25.5
10–14	187	63.1
15–19	33	11.4

*Residency*		
Busa	149	50.3
Shasha	39	13.4
Intaye	107	36.2

*Parents' education level*		
Noneducated	56	19.1
Primary level	195	65.8
Secondary level	32	11.1
College level	11	4.0

*Parents' occupational status*		
Farmer	219	73.5
Businessman	51	17.1
Civil servant	5	1.7
Daily laborer	23	7.7

*Main source of drinking water*		
Piped water	224	75.2
Well water	24	8.1
Spring water	39	13.1
River water	11	3.7

*Story water contact*		
Yes	277	93.0
No	21	7.0

*Frequency of water body contact days per week*		
3 and less time	206	69.1
4 and greater time	92	30.9

*Have awareness about schistosomiasis*		
Yes	23	7.7
No	275	92.3

*Household latrine*		
Have	271	90.9
Have no	27	9.1

**Table 2:** Prevalence of *S. mansoni* infection among schoolchildren in three primary schools of Wondo district, West Arsi Zone, South Ethiopia, 2018 (*n* = 298).

School	No. examined	No. infected (%)	No. uninfected (%)
Busa	150	14 (9.3%)	136 (90.7%)
Shasha	40	6 (15.0%)	34 (85.0%)
Intaye	108	14 (13.0%)	94 (87.0%)
Total	298	34 (11.4%)	264 (88.6%)

**Table 3:** Age distribution of *S. mansoni* among Wondo district schoolchildren who participated in this study (*n* = 298).

Age	No. examined	No. infected (%)	No. uninfected (%)
5–9	76	8 (10.5%)	68 (89.5%)
10–14	188	23 (12.3%)	165 (87.7%)
15–19	34	3 (8.8%)	31 (91.2%)
Total	298	34 (11.4%)	264 (88.6%)

**Table 4:** Intensity of schistosomiasis among Wondo district schoolchildren who participated in this study (*n* = 298). *S. mansoni*.

Intensity of infection^*∗*^	*N*	% Mean (epg)
Light 1–99	33	11.1
Moderate 100–399	1	0.3
Heavy >399	0	0
Overall	34	11.4

^*∗*^According to the WHO. ep10 mg, number of eggs per 10 mg of feaces. epg, number of eggs per gram of faeces [12].

**Table 5:** Bivariate analysis of factors associated with schistosomiasis among Wondo district schoolchildren who participated in this study (*n* = 298).

Variables	No. examined	*S. Mansoni*		OR (95% CI)	*P*
		Positive	Negative		
*Sex*					
Male	140	12	128	0.59 (0.276, 1.219)	0.150
Female	158	22	136	1	

*Age*					
5–9	76	8	68	1.216 (0.302, 4.897)	0.784
10–14	188	23	165	1.44 (0.407, 5.092)	0.571
15–19	34	3	31	1	

*Parents' education level*					
Noneducated	57	6	51	0.235 (0.054, 1.022)	0.053
Primary level	196	23	173	0.266 (0.074, 0.952)	0.042
Secondary level	33	1	32	0.062 (0.006, 0.639)	0.019
College level	12	4	8	1	

*Parents' occupation*					
Farmer	219	27	192	0.938 (0.261, 3.367)	0.921
Businessman	51	4	47	0.567 (0.116, 2.771)	0.484
Civil servant	5	0	5	0.000 (0.000, 0.000)	0.999
Daily laborer	23	3	20	1	

*Anthelminthic treatment*					
Yes	28	3	25	0.925 (0.264, 3.244)	0.903
No	270	31	239	1	
*Water source*					
Piped water	224	25	199	0.335 (0.083, 1.346)	0.123
Well water	24	1	23	0.116 (0.010, 1.280)	0.079
Spring water	39	5	34	0.392 (0.077, 1.992)	0.259
River water	11	3	8	1	

*Water contact experience*					
Have	277	26	251	0.168 (0.064, 0.444)	0.001^*∗*^
Have no	21	8	13	1	

*Water contact frequency*					
3 and less days/week	206	21	185	0.69 (0.329, 1.446)	0.325
4 and more days/week	92	13	79	1	

*Swimming*					
Yes	172	24	148	8.351 (1.931, 36.114)	0.004^*∗*^
No	105	2	103	1	

*Cloth washing*					
Yes	205	20	185	1.189 (0.458, 3.089)	0.722
No	72	6	66	1	

*Water fetching*					
Yes	133	12	121	0.921 (0.41, 2.070)	0.842
No	144	14	130	1	

*Cattle herding*					
Yes	146	16	131	1.489 (0.651, 3.408)	0.346
No	131	10	121	1	

*Travel to school*					
Yes	179	15	164	0.723 (0.319, 1.643)	0.439
No	98	11	87	1	

*Awareness on schistosomiasis*					
Have	23	8	15	1	
Have no	275	26	249	0.196 (0.076, 0.5050)	0.001^*∗*^

*Household latrine*					
Have	271	25	246	1	
Have no	27	9	18	4.92 (2.001, 12.098)	0.001^*∗*^

OR, odds ratio. CI, confidence interval. ^*∗*^Significant association (*P*, 0.025).

**Table 6:** Multivariate analysis of factors associated with *schistosomiasis* among Wondo district schoolchildren participated in this study (*n* = 298).

Variables	Schistosomiasis		*P*
	Adjusted OR	95% CI	
*Swimming*			
Yes	9.592	(1.972, 46.655)	0.005^*∗*^
No	1		
*Awareness about schistosomiasis*			
Have	1		
Have no	0.038	(0.004, 0.409)	0.007^*∗*^
*Household latrine*			
Have	1		
Have no	9.737	(2.226, 42.592)	0.003^*∗*^

OR, odds ratio. CI, confidence interval. ^*∗*^Significant key risk factors (*P*, <0.05).

## Data Availability

The SPSS output data used to support the findings of this study are available from the corresponding author upon request.
